# Exploring CP violation in the MSSM

**DOI:** 10.1140/epjc/s10052-015-3294-z

**Published:** 2015-02-21

**Authors:** Alexandre Arbey, John Ellis, Rohini M. Godbole, Farvah Mahmoudi

**Affiliations:** 1 Université de Lyon, Université Lyon 1, Centre de Recherche Astrophysique de Lyon, CNRS, UMR 5574, 69561 Saint-Genis Laval Cedex, France; 2 Ecole Normale Supérieure de Lyon, Lyon, France; 3 Theory Division, CERN, 1211 Geneva 23, Switzerland; 4 Theoretical Particle Physics and Cosmology Group, Department of Physics, King’s College London, London, WC2R 2LS UK; 5 Centre for High Energy Physics, Indian Institute of Science, Bangalore, 560012 India; 6 Institut Universitaire de France, 103 boulevard Saint-Michel, 75005 Paris, France

## Abstract

We explore the prospects for observing CP violation in the minimal supersymmetric extension of the Standard Model (MSSM) with six CP-violating parameters, three gaugino mass phases and three phases in trilinear soft supersymmetry-breaking parameters, using the CPsuperH code combined with a geometric approach to maximise CP-violating observables subject to the experimental upper bounds on electric dipole moments. We also implement CP-conserving constraints from Higgs physics, flavour physics and the upper limits on the cosmological dark matter density and spin-independent scattering. We study possible values of observables within the constrained MSSM (CMSSM), the non-universal Higgs model (NUHM), the CPX scenario and a variant of the phenomenological MSSM (pMSSM). We find values of the CP-violating asymmetry $A_\mathrm{CP}$ in $b \rightarrow s \gamma$ decay that may be as large as 3 %, so future measurements of $A_\mathrm{CP}$ may provide independent information about CP violation in the MSSM. We find that CP-violating MSSM contributions to the $B_s$ meson mass mixing term $\Delta M_{B_s}$ are in general below the present upper limit, which is dominated by theoretical uncertainties. If these could be reduced, $\Delta M_{B_s}$ could also provide an interesting and complementary constraint on the six CP-violating MSSM phases, enabling them all to be determined experimentally, in principle. We also find that CP violation in the $h_{2,3} \tau ^+ \tau ^-$ and $h_{2,3} {\bar{t}} t$ couplings can be quite large, and so may offer interesting prospects for future $pp$, $e^+ e^-$, $\mu ^+ \mu ^-$ and $\gamma \gamma$ colliders.

## Introduction

The minimal supersymmetric extension of the Standard Model (MSSM) contains many possible sources of CP violation beyond the Kobayashi–Maskawa phase of the Standard Model and the strong CP phase. These additional sources of CP violation arise from the soft supersymmetry-breaking terms in the low-energy effective Lagrangian, and include phases in the gaugino masses, the trilinear scalar couplings and the sfermion mass matrices. However, the Kobayashi–Maskawa phase accounts very well for the CP-violating effects seen in the $K^0$ system and in $B$ meson decays, and no other violations of CP have been observed despite, for example, sensitive experimental searches for electric dipole moments (EDMs). Thus, it might be tempting to suggest that the extra MSSM sources of CP violation are absent. On the other hand, experimental upper limits still allow considerable scope for additional CP-violating effects in, for example, $B^0_s$ mixing, and some additional source of CP violation is needed to explain the cosmological baryon asymmetry, which might be due to these MSSM phases. For these reasons, there have been many studies of possible MSSM CP-violating effects in experimental observables, and powerful phenomenological tools have been developed for calculating these effects.

In view of the success of the Cabibbo–Kobayashi–Maskawa (CKM) model in describing flavour mixing and CP violation in the quark sector, it is often assumed that the strong CP phase is negligibly small for some reason, and that flavour and CP violation for squarks is generated by the CKM mixing in the quark sector, the hypothesis of minimal flavour violation (MFV). However, even in this case there remain several additional sources of CP violation in the MSSM, namely the phases in the gaugino masses and the trilinear couplings. One is thus led to consider the maximally CP-violating, minimal flavour-violating (MCPMFV) model that contains six CP-violating phases beyond the Kobayashi–Maskawa phase: three phases $\Phi _{1,2,3}$ in the masses of the U(1), SU(2) and SU(3) gauginos, and three phases $\Phi _{A_{t,b,\tau }}$ in the trilinear soft supersymmetry-breaking couplings $A_{t,b,\tau }$ of the third-generation stop, sbottom and stau sfermions, respectively.^1^ In this study, we allow the six CP-violating phases to vary independently in all the scenarios considered. Predictions of the MCPMFV scenario for CP-violating observables such as the CP-violating asymmetry in $b \rightarrow s \gamma$ decay, $A_\mathrm{CP}$, the CP-violating phase in $B_s$ mixing, $\phi _s$, and EDMs have been considered in [1, 2], and possibilities for probing these CP-violating phases through the polarisation of third-generation fermions, $t$ and $\tau$, produced in the decays of the corresponding sfermions have also been explored [3].

It might be thought that the MSSM phases $\Phi _{1,2,3,t,b,\tau }$ must necessarily be small, in view of the stringent upper limits on several EDMs shown in Table 1. However, this is not necessarily the case, since there are four main independent EDM constraints on what is, a priori, a 6-dimensional space of CP-violating MSSM phases, so there are in principle ‘blind directions’ corresponding to combinations of phases that do not ‘see’ the EDM constraints. In principle, individual phases could be large along these directions, as discussed in [4] for example, and could have significant effects on other CP-violating observables such as $A_\mathrm{CP}$ and $\phi _s$.^2^

**Table 1:** The 95 % CL upper limits on EDMs used as constraints in this study. The present experimental upper bound on the EDM of the muon, $1.9\times 10^{-19}$ e.cm [10], provides only a very weak constraint that is not competitive with the other EDM constraints in the models discussed here

EDM	Upper limit (e.cm)	Reference
Thallium	$1.3\times 10^{-24}$	[6]
Mercury	$3.5\times 10^{-29}$	[7]
Neutron	$4.7\times 10^{-26}$	[8]
Thorium monoxide	$1.1\times 10^{-28}$	[9]

A brute force way to study this possibility would be to sample randomly the 6-dimensional space of CP-violating MSSM phases, but this is not the most efficient procedure to explore the possible magnitudes of CP-violating effects in the MSSM. If one wishes to generate a large sample of parameter sets that respect other phenomenological constraints such as those from the flavour, Higgs and dark matter sectors, one would prefer to optimise the search for MSSM scenarios with maximal CP violation. A geometric approach to this problem was proposed in [2] and used to analyse the impacts of three EDM constraints in certain specific benchmark MSSM scenarios.

In this paper we adapt and extend this geometric approach to study systematically the possible magnitudes of CP-violating effects in light of the updated EDM constraints shown in Table 1. The inclusion of a fourth EDM constraint requires a slight extension of the analysis based on three EDMs made in [2], as we discuss in Sect. 2. Also, the geometric approach was originally formulated as a linear expansion around the CP-conserving limit, whereas we are interested in the largest possible values of the CP-violating phases. Accordingly, here we extend the approach using an iterative procedure, finding an initial ‘blind direction’ as in [2], then choosing a CP-violating point along that direction with non-zero phases, and then repeating the geometrical optimisation in a new linear approximation around this CP-violating point, as also discussed in Sect. 2. In Sect. 3 we then apply the geometric approach to four variants of the MSSM, a best-fit scenario [11, 12] within the constrained MSSM (CMSSM) in which the soft supersymmetry-breaking parameters are constrained to be universal at the GUT scale (apart from the CP-violating phases), a generalisation of this model in which the soft supersymmetry-breaking contributions to the two Higgs doublet masses are allowed to vary independently (NUHM2), a version of the CPX scenario defined in [13] that is modified to be in agreement with the LHC results, and the phenomenological MSSM (pMSSM) [14], in which extrapolation to the GUT scale is ignored and universality is not imposed.^3^ In each case, in addition to the EDM constraints in Table 1, we also consider the relevant constraints from flavour physics, from the measured properties of the known Higgs boson and searches for other MSSM Higgs bosons, and upper limits on the cosmological density of dark matter and the direct detection of dark matter via scattering on nuclei.

We focus, in particular, on four possible signatures of MSSM CP violation: the possibility that there might be another neutral Higgs boson lighter than the one already discovered by ATLAS and CMS, the CP-violating asymmetry in $b \rightarrow s \gamma$ decay, $A_\mathrm{CP}$, and the non-Standard-Model contribution to the $B_s$ meson mixing parameter, $\Delta M_{B_s}$, and CP-violating couplings of the heavier neutral Higgs bosons. We find that, although a neutral MSSM Higgs boson lighter than that discovered would be consistent with the EDM constraints, it is excluded by the available limits on other Higgs bosons, notably the absence of a light charged Higgs boson. Secondly, we find that values of $A_\mathrm{CP} \lesssim 3$ % are allowed by the EDMs and other constraints in some of the MSSM scenarios studied. This opens up the possibility that $A_\mathrm{CP}$ could be significantly larger than in the Standard Model, providing a signature of CP-violating MSSM. Conversely, if a non-zero value of $A_\mathrm{CP}$ were not to be found in future experiments, this could provide a constraint on CP violation in the MSSM that is independent of, and complementary to, those from EDMs. Thirdly, in the case of $\Delta M_{B_s}$, we find that it could also provide an independent constraint on the CP-violating MSSM if the theoretical uncertainties could be reduced, thereby enabling in principle a complete determination of all the phases for fixed values of the CP-conserving MSSM parameters. Fourthly, we also find that CP violation in the $h_{2,3} \tau ^+ \tau ^-$ and $h_{2,3} {\bar{t}} t$ couplings can be quite large, and may offer interesting prospects for future $pp$, $e^+ e^-$ and $\mu ^+ \mu ^-$ experiments.

## Method

In this section we outline our approach to sampling the parameter spaces of MSSM scenarios while respecting the four EDM constraints in Table 1. Since the EDM constraints are quite strong, they effectively reduce the dimensionality of any MSSM scenario by four. The challenge is to sample efficiently this subspace of codimension four, so as to assess how large any other CP-violating observable may be. Moreover, the thorium monoxide EDM constraint on the electron EDM is now so strong that we have designed a new method to sample effectively the parameter space. We do this by adapting and extending the geometric approach proposed in [2]. In the first subsection we discuss how the approach may be modified to take into account four EDM constraints, in the following subsection we describe an extension of the analysis beyond the small-phase approximation, and in the third subsection we summarise our sampling algorithm.

### Geometric approach to maximizing a CP-violating observable with four EDM constraints

Initially, we consider the four EDMs $E^{a,b,c,d}$ of Table 1 in the small-phase approximation,^4^ where

1 $$\begin{aligned} E^i \simeq {\varvec{\Phi }}.{\mathbf E}^i, \end{aligned}$$

with ${\varvec{\Phi }}\equiv \Phi _\alpha = \Phi _{1,2,3,t,b,\tau }$ and ${\mathbf E}^i \equiv \partial E^i/\partial {\varvec{\Phi }}$ (i.e., $E^i_\alpha \equiv \partial E^i/\partial \Phi _\alpha$). The ${\varvec{\Phi }}$ subspace of codimension four is spanned by the following quadruple exterior product:

2 $$\begin{aligned} A_{\alpha \beta \gamma \delta }=E^a_{[ \alpha } \, E^b_\beta \, E^c_\gamma \, E^d_{\delta ]} \end{aligned}$$

where the symbols $[\ldots ]$ denote antisymmetrisation of the enclosed indices. This subspace is a 2-dimensional plane, as in the simple example in Section 2.1 of [2]. We now consider some CP-violating observable $O$ whose dependence on the phases $\Phi _\alpha$ is given in the small-phase approximation by ${\mathbf O} \equiv \partial O/\partial {\varvec{\Phi }}$ (i.e., $O_\alpha \equiv \partial O/\partial \Phi _\alpha$). One can then define the vector

3 $$\begin{aligned} B_\mu \equiv \epsilon _{\mu \nu \lambda \rho \sigma \tau } \, O_\nu \, E^a_\lambda \, E^b_\rho \, E^c_\sigma \, E^d_\tau \end{aligned}$$

that characterises a direction in the space of CP-violating phases where there is no contribution to the observable $O$, nor to the EDMs. The EDM-free direction that optimises $O$ is clearly orthogonal to $B_\mu$ as well as to the EDM vectors $E^{a,b,c,d}_\alpha$. As such, it is characterised by the six-vector

4 $$\begin{aligned} \Phi _\alpha&= \epsilon _{\alpha \beta \gamma \delta \mu \eta } \, E^a_\beta \, E^b_\gamma \, E^c_\delta \, E^d_\mu \, B_\eta \nonumber \\&= \epsilon _{\alpha \beta \gamma \delta \mu \eta } \, \epsilon _{\eta \nu \lambda \rho \sigma \tau } \, E^a_\beta \, E^b_\gamma \, E^c_\delta \, E^d_\mu \, O_\nu \, E^a_\lambda \, E^b_\rho \, E^c_\sigma \, E^d_\tau , \end{aligned}$$

with an unknown normalisation factor.

### Iterative geometric approach

The linear geometric approach described above and used in [2] entails choosing a sample of points in the MSSM scenario of interest, fixing the phases to $0^{\circ }$ or ${\pm }180^{\circ }$ for each scan point. Next one computes the optimal direction using the above geometric approach, and then one chooses randomly sets of phases along this direction. This is suitable as long as the phases are small, but we are also interested in the possibilities for large phases.

Here we use an iterative approach to extend and improve the efficiency of the linear geometric approach. After fixing the phases to $0^{\circ }$ or ${\pm }180^{\circ }$ and computing the favoured direction with the geometric approach as discussed above, we move by $20^{\circ }$ along the favoured direction, and then recompute the favoured direction at this new position. This procedure is then iterated up to $100^{\circ }$.

### Sampling strategy

We have generated several million points in each of the MSSM scenarios studied in the next section. Among those points, we have retained only those for which one of the neutral Higgs bosons has a mass in the range 121–129 GeV (corresponding to the measured value ${\simeq }125$ GeV with a generous theoretical uncertainty), and we require the LSP to be the lightest neutralino. In addition, we impose the LEP and Tevatron SUSY mass limits and require squarks and the gluino to have masses above 500 GeV as a conservative implementation of the LHC SUSY limits. Although the LHC SUSY search limits are stronger in more constrained MSSM scenarios, they become weaker in more general scenarios such as the pMSSM [16–18]. For consistency, here we apply the same loose constraints on the squark and gluino masses in all studied scenarios. Other constraints, such as those imposed by heavy-flavour, Higgs and direct dark matter measurements, are imposed at later stages in the analyses.

The SUSY mass spectra and couplings, as well as the EDM constraints, are computed with CPsuperH [19–21]. The thorium monoxide EDM is calculated using the following formula [22]:

5 $$\begin{aligned}&d_\mathrm{ThO} [e.\mathrm{cm}]/{\mathcal {F}}_\mathrm{ThO} = d_e [e.\mathrm{cm}] +1.6\nonumber \\&\quad \times 10^{-21}[e.\mathrm{cm}] \; C_S\;\mathrm{TeV}^2+\cdots \!, \end{aligned}$$

where $d_e$ is the electron EDM and $C_S$ the coefficient of the CP-odd electron nucleon interaction, which is also present in the thallium EDM. The left hand side of Eq. (5) is the quantity on which experimental constraints are provided currently [22]. Flavour constraints are calculated with SuperIso [23, 24] and CPsuperH. For the calculation of the dark matter relic density we used SuperIso Relic [25] and micrOMEGAs [26–28], and the later is also used for the calculation of scattering cross sections for dark matter direct detection. Finally, we use HiggsBounds [29] to assess the viability of the model points in view of the Higgs constraints.

## Studies of MSSM scenarios

We now apply the approach described above to several representative MSSM scenarios.

### The CMSSM

We first consider the CMSSM, in which the soft supersymmetry-breaking parameters $m_0, m_{1/2}$ and $A$ are each constrained to have universal values at an input grand-unification scale. This model is often analysed assuming some fixed value of $\tan \beta$, the ratio of Higgs v.e.v.s. Generalizing the usual CMSSM set-up, here we vary the 6 MSSM CP phases independently in order to allow more flexibility and a closer comparison with the other MSSM scenarios. Our starting-point here is one of the best-fit CMSSM points found recently in a global analysis [11] of the $m_0, m_{1/2}, A, \tan \beta$ parameter space for the Higgsino mixing parameter $\mu > 0$, neglecting all the possible MSSM sources of CP violation.^5^ This point has

6 $$\begin{aligned}&m_0 =670~\mathrm{GeV},\quad m_{1/2}=1040~\mathrm{GeV},\quad A=3440~\mathrm{GeV},\nonumber \\&\quad \tan \beta =21. \end{aligned}$$

We use this point as a base for the geometric approach using the EDM limits in Table 1, treating the CP asymmetry in $b \rightarrow s \gamma$, $A_\mathrm{CP}$, as the observable to be maximised, in a follow-up of the study presented in [15]. We have generated more than 600000 sets of phases along the favoured direction, and have found that about half of them pass the EDM constraints, which shows that the method is very efficient.

**Fig. 1:**
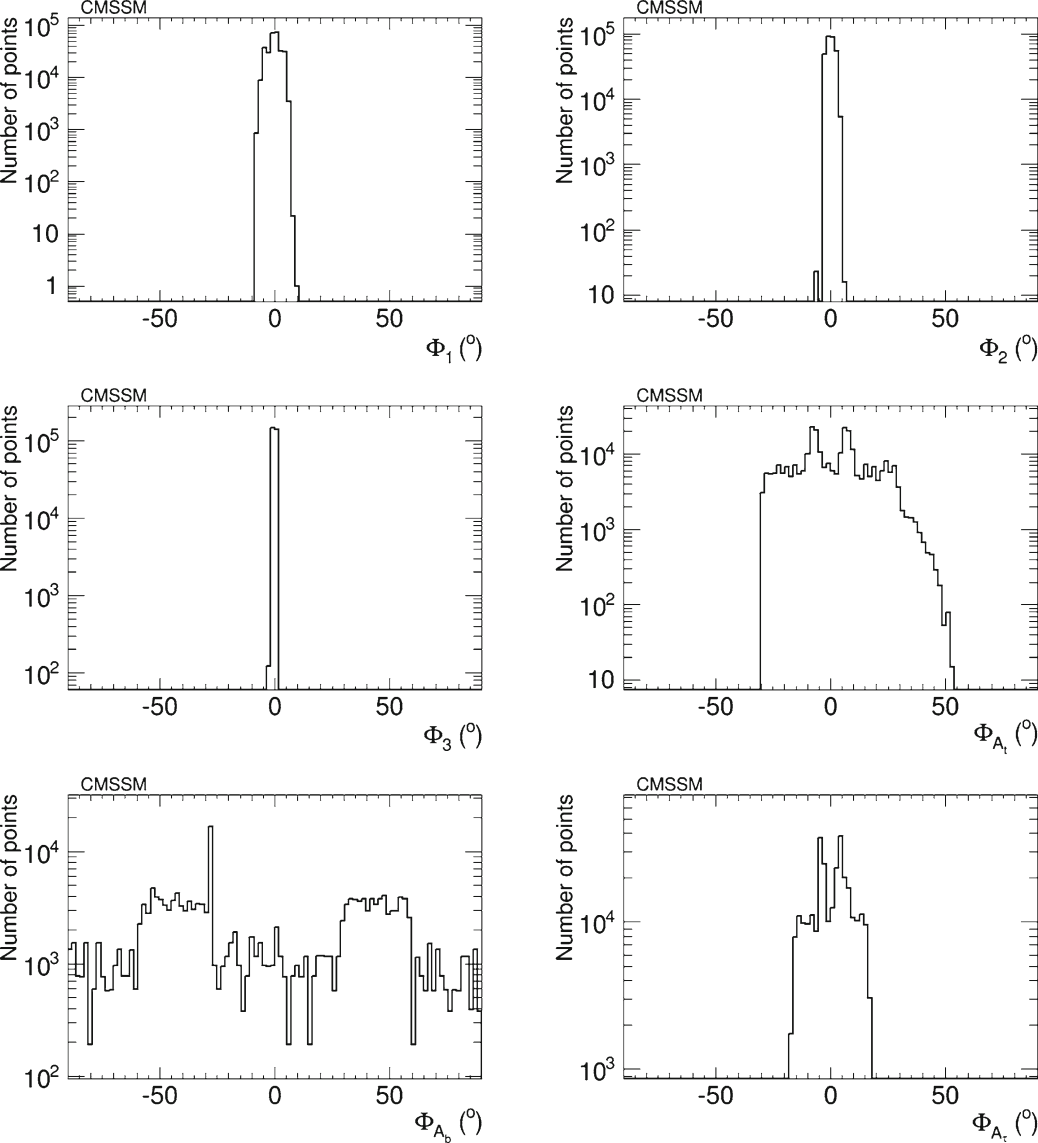
Sampling of the CP-violating phases $\Phi _\alpha$ in the best-fit CMSSM scenario (6) generated in the iterative geometric approach, imposing the EDM limits as well as constraints from the cosmological cold dark matter relic density and upper limits on direct detection, from flavour physics and from Higgs searches

Figure 1 shows the distributions of the six CP-violating phases $\Phi _\alpha$ obtained from our sampling. The reader should bear in mind that these distributions have no ‘probability’ or ‘likelihood’ interpretation but only indicate how our iterative geometric procedure samples large values of the phases. We see that the effectiveness of the procedure differs significantly for different phases. We see that large values of $\Phi _{A_b}$ are relatively well sampled, whereas only intermediate $\Phi _{A_t}$ and $\Phi _{A_\tau }$ values can be reached, and we find no parameter sets with $\Phi _{1,2,3}$ substantially different from zero. This is because for the CMSSM best-fit point (6) it is not possible to cancel the contributions of the phases to all the EDMs simultaneously.

**Fig. 2:**
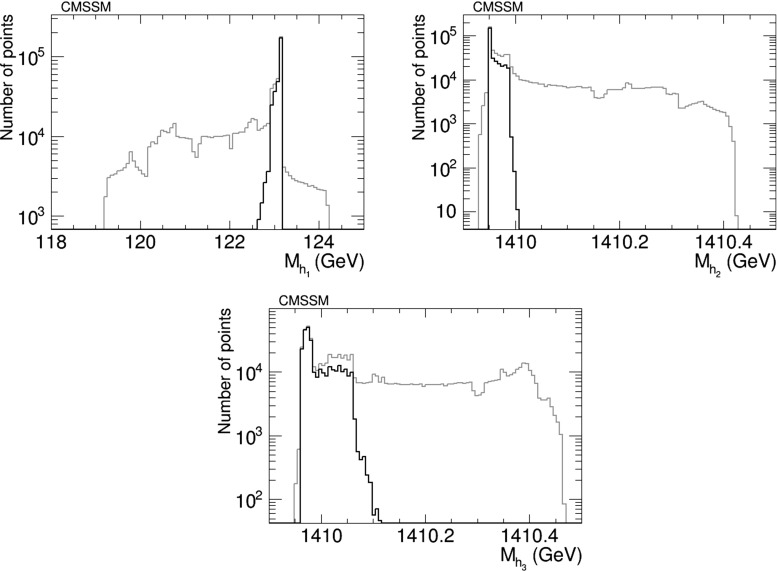
The distributions of the *three* Higgs masses $M_{h_1},M_{h_2},M_{h_3}$ in the CP-violating CMSSM before (*grey*) and after (*black*) applying the EDM constraints using the geometric approach described in the text, assuming the best-fit values of $m_0, m_{1/2}, A$ and $\tan \beta$ (6) found in a global analysis

Figure 2 displays the results of this scan of the CP-violating CMSSM for the masses of the three neutral Higgs bosons $M_{h_1},M_{h_2},M_{h_3}$. The Higgs masses all lie in narrow ranges around their nominal values at the best-fit point in the CP-conserving CMSSM, namely $M_h = 123$ GeV, $M_A \simeq M_H = 1410$ GeV. In view of the theoretical uncertainties in calculating the Higgs masses for any specific set of CMSSM inputs, measuring Higgs masses would not constrain usefully the CP-violating parameters at the CMSSM best-fit point.

**Fig. 3:**
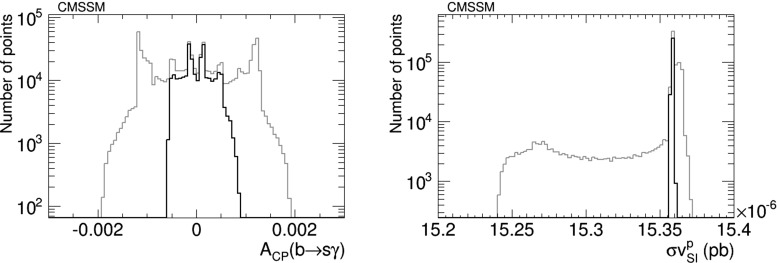
The distributions of (*left*) the CP asymmetry in $b\rightarrow s\gamma$, $A_\mathrm{CP}$, in the CMSSM and (*right*) the spin-independent scattering cross section for neutralino scattering on protons, found before (*grey*) and after (*black*) applying the EDM constraints using the geometric approach described in the text and assuming the best-fit values of $m_0, m_{1/2}, A$ and $\tan \beta$ (6) found in a global analysis [11, 12]

Figure 3 displays the results of this CMSSM scan for the CP asymmetry in $b\rightarrow s\gamma$, $A_\mathrm{CP}$, (left) and the spin-independent neutralino–proton scattering cross section $\sigma _\mathrm{SI}^p v$ (right). We find in this model values of $A_\mathrm{CP} \ll 10^{-3}$, which are considerably below the current and prospective experimental sensitivities. We conclude that the prospects for discovering the $A_\mathrm{CP}$ signature of CP violation in this particular CMSSM scenario are not good. Also, the spread in the values of $\sigma _\mathrm{SI}^p v$ is quite small, and much smaller than the theoretical uncertainties related to hadronic matrix elements and the astrophysical uncertainties in the local dark matter density, so this observable is also not a promising one for the CP-violating CMSSM.

We have also studied the possibility of a signature in $B_s$ meson mass mixing, with discouraging results. We have found that the new physics contribution, $\Delta M^\mathrm{NP}_{B_s}$ is always very small, namely ${\sim } 0.1$/ps, which is far below any prospective reduction in the uncertainty in the theoretical calculation of the contribution from the Standard Model [30]. Moreover, after applying the EDM constraints the CP-violating CMSSM contribution is forced to be exceedingly close to the value in the CP-conserving CMSSM.

**Fig. 4:**
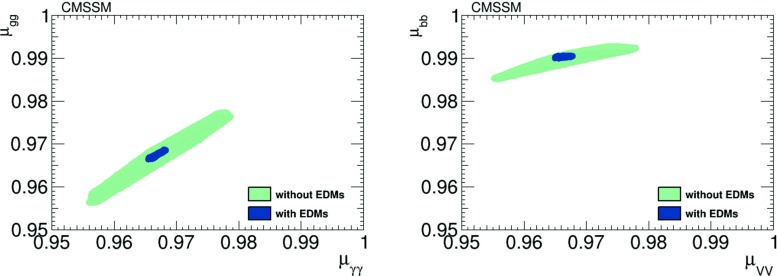
Scatter plots of the $h_1$ signal strengths in the best-fit CMSSM scenario (6) in the CP-violating limit before (*green dots*) and after (*blue dots*) imposing the EDM constraints. The *left panel* displays a strong linear correlation between $\mu _{\gamma \gamma }$ and $\mu _{gg}$, and the *right panel* displays the correlation between $\mu _{VV}$ and $\mu _{{\bar{b}}b}$

In Fig. 4 we show scatter plots of $h_1$ signal strengths $\mu _X$ (normalised relative to the Standard Model values) in the best-fit CMSSM scenario (6) with non-zero CP-violating phases before (green dots) and after (blue dots) the EDM constraints. We see that the CP-violating case expands the ranges of these observables found already in the CP-conserving case, in particular after imposing the EDM constraints. However, these expanded ranges all lie well within the current experimental uncertainties. In the left panel we see a strong, almost linear correlation between $\mu _{\gamma \gamma }$ and $\mu _{gg}$, which becomes milder in the right panel, between $\mu _{VV}$ and $\mu _{{\bar{b}}b}$. The signal strengths are close to but smaller than unity.

We emphasise that the Higgs couplings to fermions provide the most unambiguous probe of its CP properties, even more so when there is CP mixing, as the Higgs may couple to the CP-even and CP-odd fermion states in a democratic manner. In cases of the $h_{i}VV$ couplings, there are two effects of CP mixing in the Higgs sector. One is a reduction in the strength of coefficient of the $g_{\mu \nu }$ term in the $h_{i} VV$ vertex and thus in the rates. The second is the simultaneous presence of the CP-even and CP-odd tensor structures in the vertex. The coefficient of the CP-odd term in the $h_{i} VV$ vertex involving the $\epsilon _{\mu \nu \rho \lambda }$ tensor is by necessity small, as it is always loop-induced. Reduction in the production rates is reflected in signal strengths, but these, while currently providing the best available information, are necessarily ambiguous, as there are other mechanisms that may lead to the rate modification. On the other hand, since the fermions couple democratically to the CP-even and CP-odd parts of the Higgs couplings, ascertaining the simultaneous presence of ${\bar{f}} f h_{i}$ and ${\bar{f}} \gamma _5 f h_{i}$ terms in the vertex through various angular distributions and kinematic variables is unambiguous.

We have therefore analysed the prospects for CP violation in the couplings of the neutral Higgs bosons to $\tau ^+ \tau ^-$ and ${\bar{t}} t$, by calculating the quantities $\phi ^{h_i}_\tau$ and $\phi ^{h_i}_t$ for $i = 1, 2, 3$, which are expressed in terms of the corresponding pseudoscalar and scalar couplings by

7 $$\begin{aligned} \tan \phi ^{h_i}_\tau \equiv \frac{g_P^{h_i \tau \tau }}{g_S^{h_i \tau \tau }},\quad \tan \phi ^{h_i}_t \equiv \frac{g_P^{h_i {\bar{t}} t}}{g_S^{h_i {\bar{t}} t}}. \end{aligned}$$

**Fig. 5:**
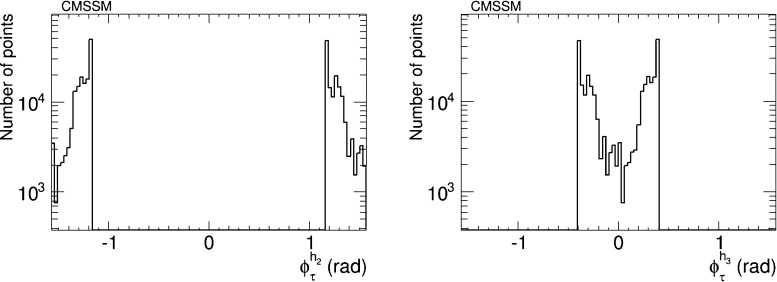
The distributions of (*left*) the CP-violating phase $\phi _\tau ^{h_2}$ in $h_2 \tau \tau$ couplings and (*right*) the CP-violating phase $\phi _\tau ^{h_3}$ in $h_3 \tau \tau$ couplings in the CMSSM, found after applying the EDM constraints using the geometric approach described in the text and assuming the best-fit values of $m_0, m_{1/2}, A$ and $\tan \beta$ (6) found in a global analysis [11, 12]

**Fig. 6:**
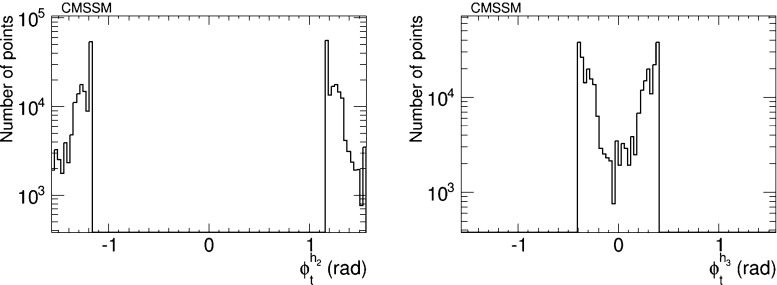
The distributions of (*left*) the CP-violating phase $\phi _t^{h_2}$ in $h_2 {\bar{t}} t$ couplings and (*right*) the CP-violating phase $\phi _t^{h_3}$ in $h_3 {\bar{t}} t$ couplings in the CMSSM, found after applying the EDM constraints using the geometric approach described in the text and assuming the best-fit values of $m_0, m_{1/2}, A$ and $\tan \beta$ (6) found in a global analysis [11, 12]

After imposing the EDM constraints, we find that the phases for the $h_1$ couplings are very small, ${\lesssim } 0.02$ radians. On the other hand, the phases for the $h_2$ and $h_3$ couplings may be quite large, as seen in Figs. 5 and 6, respectively. The $h_2$ couplings have phases close to ${\pm } \pi$, corresponding to a mainly CP-odd state, while the $h_3$ couplings are close to 0 corresponding to a mainly CP-even state. A detailed discussion of the prospects for measuring these phases at the LHC and/or future colliders lies beyond the scope of this work. Clearly, any such future analysis would need to take into account the near-degeneracy of the $h_2$ and $h_3$ bosons, as seen in Fig. 2, whose implications would be different for $pp$, $e^+ e^-$, $\mu ^+ \mu ^-$ and $\gamma \gamma$ colliders.

We limit ourselves to pointing out a few of these. In the case of the light Higgs, associated production of Higgs with a ${\bar{t}} t$ pair or a single $t$ or ${\bar{t}}$ can be used for this [31, 32]. However, in our case since it is the heavier Higgses that have the larger CP violation, associated production may not be the best way, but decays of the Higgs into a $\tau$ pair (or even into a ${\bar{t}} t$ pair if the Higgs is heavy enough) and analysis of the spins of the decay $\tau /t$ can be used at $\gamma \gamma$ colliders [33–36] and even at the LHC [37–39]. The method of Ref. [37] is particularly promising when the $h_{2,3}$ are degenerate.

### NUHM2

We now use the iterative geometric approach with four EDM constraints to analyse CP violation in the NUHM2 scenario, in which the gaugino masses, trilinear couplings and soft supersymmetry-breaking contributions to the squark and slepton masses $M_{\tilde{f}}$ are universal, but those to the two Higgs doublets are allowed to vary independently. The freedom in these two parameters can be traded via the electroweak vacuum conditions for free values of $\mu$ and a heavy Higgs mass parameter: to avoid complications with the three-way CP-violating mixing in the neutral sector, we take this second free parameter to be $m_{H^\pm }$. We perform a random scan over the following ranges of the NUHM2 mass parameters:

8 $$\begin{aligned}&M_1 =M_2=M_3=m_{1/2} \in [50,3000]~\mathrm{GeV}\!, \nonumber \\&\quad A_0 \in [0,10000]~\mathrm{GeV}, \nonumber \\&M_{\tilde{f}}=m_0 \in [50,3000]~\mathrm{GeV},\quad m_{H^\pm } \in [1,2000]~\mathrm{GeV},\nonumber \\&\quad \mu \in [{-}2000,2000]~\mathrm{GeV}, \end{aligned}$$

with $\tan \beta \in [1,60]$ and varying the six phases $\Phi _\alpha$ independently as before, using the geometrical approach to seek maximal values of $A_\mathrm{CP}$.

**Fig. 7:**
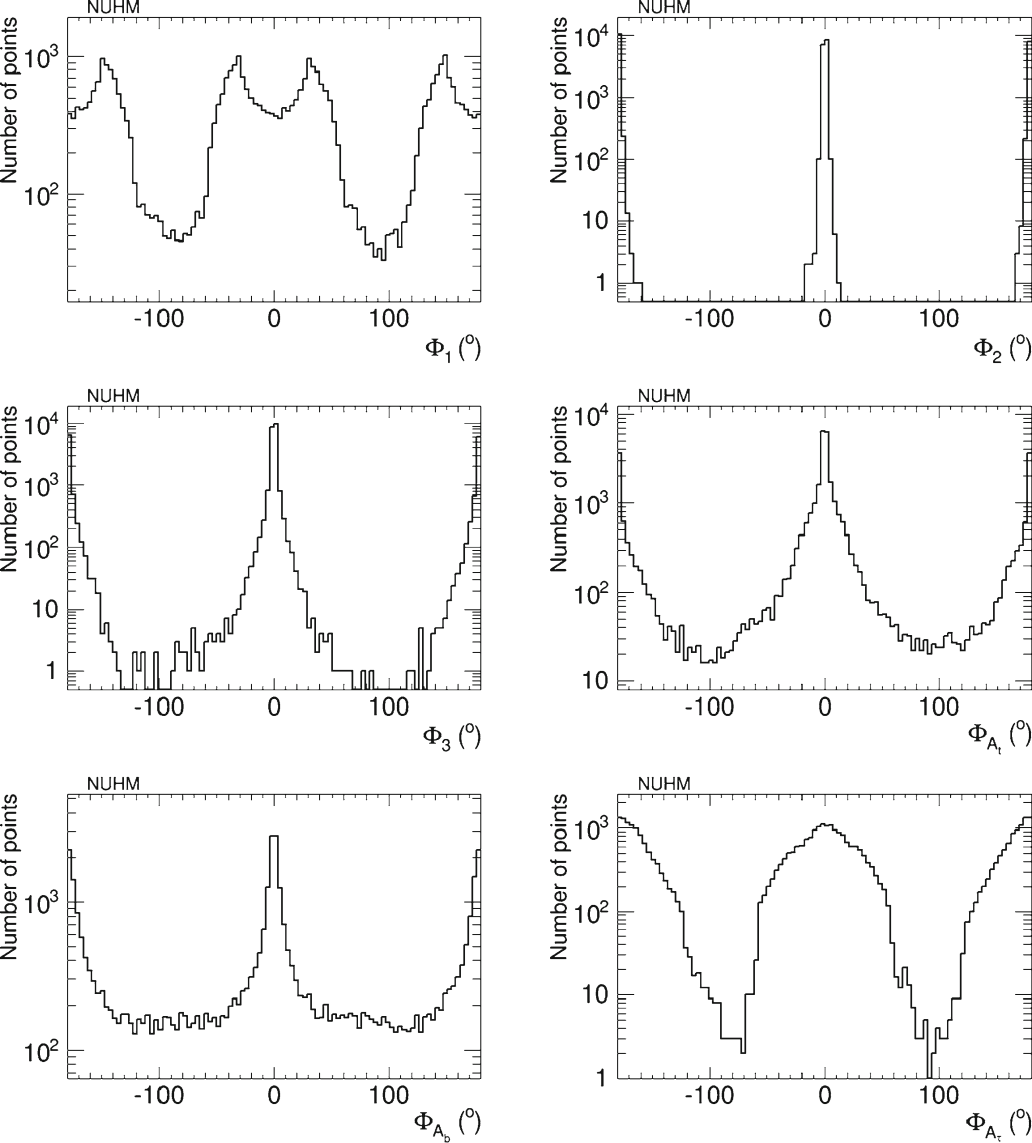
Sampling of the CP-violating phases $\Phi _\alpha$ in the NUHM2 scenario generated in the iterative geometric approach, imposing the EDM and other constraints

Figure 7 displays the samples of the six CP-violating phases $\Phi _\alpha$ obtained in our analysis. We see that our iterative geometrical approach enables us to sample effectively large values of $\Phi _1, \Phi _{A_t}$ and $\Phi _{A_b}$, whereas large values of $\Phi _3$ and $\Phi _{A_\tau }$ are sampled less effectively, and we do not find large values of $\Phi _2$. Again, we emphasise that these distributions do not have any ‘probability’ or ‘likelihood’ interpretation. However, the absence of large values of $\Phi _2$ indicates that there is no way to cancel the contributions of this and the other phases to all the EDMs simultaneously.

**Fig. 8:**
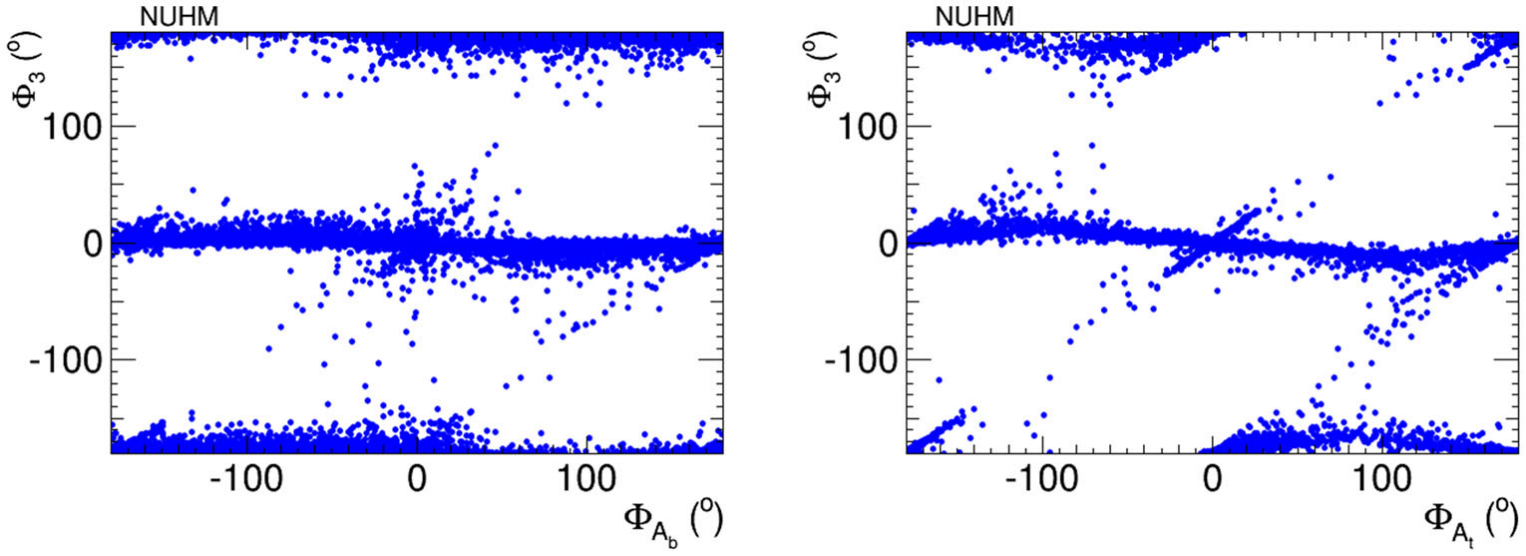
Correlations of $\Phi _3$ with $\Phi _\mathrm{{A_b}}$ (*left panel*) and $\Phi _3$ with $\Phi _\mathrm{{A_t}}$ (*right panel*) imposed by the EDM constraints in the NUHM2 scenario

Figure 8 provides a visualisation of the cancellations that are required to respect the EDM constraints. In the left panel we see the correlation these constraints impose between $\Phi _3$ and $\Phi _{A_t}$, and in the right panel the correlation between $\Phi _3$ and $\Phi _{A_b}$. In both cases we see diagonal features corresponding to close correlations, but we also see populations of points with large phases, e.g., in the neighbourhood of $(\Phi _{A_t}, \Phi _3) \sim (90^{\circ }, -90^{\circ })$ in the left panel, and extending to $(\Phi _{A_b}, \Phi _3) \sim (-90^{\circ }, -90^{\circ })$ in the right panel. These examples serve as reminders that the EDM constraints do not require all the CP-violating phases to be small simultaneously.

**Fig. 9:**
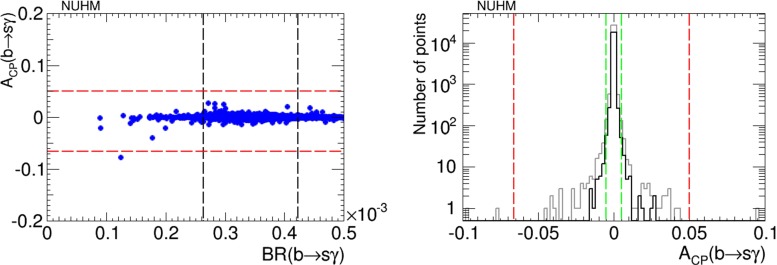
*Left panel* Scatter plot of the branching ratio for $b\rightarrow s\gamma$ decay versus its CP-violating asymmetry, $A_\mathrm{CP}$, in the NUHM2 scenario. *Right panel* Histogram of $A_\mathrm{CP}$ in the NUHM2, imposing only the Higgs mass and EDM cuts (*grey* full sample, *black* points satisfying the EDM constraints). The *vertical red dashed lines* represent the present experimental limits, and the *vertical green dashed lines* represent the prospective future improvement in the sensitivity to $A_\mathrm{CP}$ by a factor of 10

The iterative geometric approach was designed to find the maximal values of the CP-violating asymmetry in $b \rightarrow s \gamma$ decays, $A_\mathrm{CP}$, that are compatible with the EDM constraints. We see in the left panel of Fig. 9 that values of $A_\mathrm{CP} \lesssim 2$ % can be found in the NUHM2 for values of the $b \rightarrow s \gamma$ branching ratio lying within the experimentally allowed range. The right panel of Fig. 9 displays a histogram of these results for the NUHM2 (grey: full sample, black: points satisfying the EDM constraints). The present experimental constraints on $A_\mathrm{CP}$ are shown as vertical red dashed lines [40], and the vertical green dashed lines represent the possible future improvement in the experimental sensitivity by a factor of 10, corresponding to the prospective Belle II sensitivity [41]. We see that there are CP-violating NUHM2 models that could be explored with such an improvement: the EDM constraints do not exclude an observable value of $A_\mathrm{CP}$, and such a measurement would provide additional information on CP violation within the NUHM2.

**Fig. 10:**
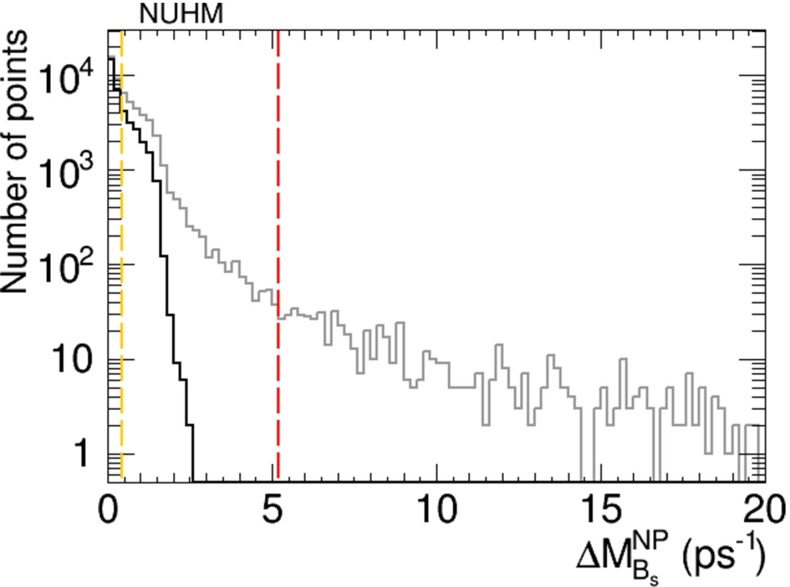
Histogram of the possible new physics contribution to $B_s$ mixing, $\Delta M^\mathrm{NP}_{B_s}$, in the NUHM2 scenario. The *grey histogram* is for points satisfying the Higgs mass and other constraints, and the *black histogram* is for points that also satisfy the EDM constraints. The *vertical red dashed line* is the present experimental upper limit on $\Delta M^\mathrm{NP}_{B_s}$, and the *vertical yellow dashed line* shows the potential of a reduction in the current theoretical uncertainty in the Standard Model by a factor of 10

We have also calculated the possible new physics contribution to $B_s$ meson mass mixing, $\Delta M^\mathrm{NP}_{B_s}$, in the NUHM2 scenario, as shown in Fig. 10. The grey histogram is for the full sample of NUHM2 points satisfying the Higgs mass and other constraints, and the black histogram is for points that also satisfy the EDM constraints. The present experimental upper limit on $\Delta M^\mathrm{NP}_{B_s}$ is shown as the vertical red dashed line [40]. The vertical yellow dashed line in Fig. 10 represents the possible sensitivity if the theoretical uncertainty in the Standard Model contribution to $B_s$ mixing could be reduced by a factor of 10 thanks to improved lattice calculations. In this case, many of the viable NUHM2 models (indicated by the black histogram) could be explored.

**Fig. 11:**
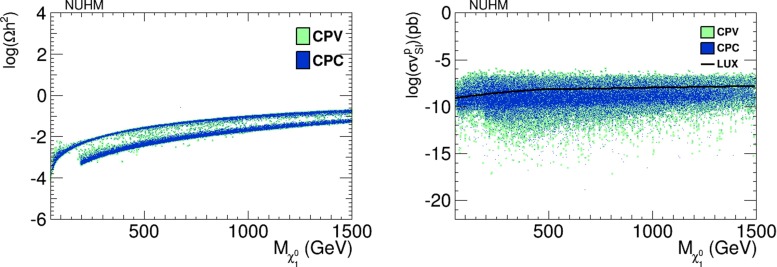
*Left panel* Scatter plot of the dark matter relic density as a function of the neutralino mass in the NUHM2 scenario. *Right panel* Scatter plot of the spin-independent dark matter scattering cross section $\sigma _\mathrm{SI}^p v$ as a function of the neutralino mass in the NUHM2 scenario. In both panels, CP-conserving parameter choices are denoted by *blue dots*, and CP-violating parameter choices by *green dots*

We have not imposed a priori consistency with the cosmological constraints on the relic LSP density $\Omega _{\chi } h^2$ and the spin-independent dark matter scattering cross section $\sigma _\mathrm{SI}^p v$. As we see in the left panel of Fig. 11, the values of the relic density for the CP-violating NUHM2 (green points) are very similar to those in the CP-conserving version (blue points), and they are generally within the range allowed for a supersymmetric contribution to the dark matter density. The right panel of Fig. 11 shows that the values of $\sigma _\mathrm{SI}^p v$ are also rather similar, with some differences for low cross-section values well below the experimental upper limit from LUX [42], which is shown as the black solid line.

**Fig. 12:**
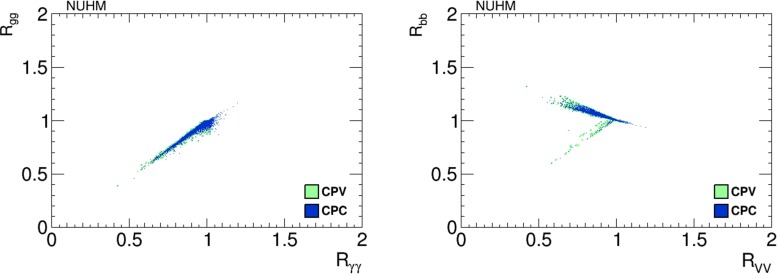
Scatter plots of branching ratios, normalised to the Standard Model values, for decays of the lightest Higgs boson, $h_1$, in the NUHM2 scenario in the CP-violating limit $\Phi _\alpha = 0$ (*blue dots*) and in the CP-violating sample (*green dots*). The *left panel* displays a linear correlation between $R_{\gamma \gamma }$ and $R_{gg}$, and the *right panel* displays a bimodal correlation between $R_{VV}$ and $R_{{\bar{b}}b}$

Figure 12 shows scatter plots of values of $h_1$ branching ratios in the NUHM2 scenario. The left panel displays $(R_{\gamma \gamma }, R_{gg})$ and the right panel displays $(R_{VV}, R_{{\bar{b}}b})$. The blue dots are CP-conserving parameter choices with $\Phi _\alpha = 0$, and the green dots are from a scan of CP-violating points with $\Phi _\alpha \ne 0$. We note in the left panel a strong correlation between $R_{\gamma \gamma }$ and $R_{gg}$, which may be either much smaller than in the Standard Model or somewhat larger, which is due to the variation of the Higgs width induced by a modification of the Higgs to ${\bar{b}}b$ branching fraction.^6^ We see in the right panel that a large reduction in $R_{VV}$ is also possible, which may be accompanied by values of $R_{{\bar{b}}b}$ that are either larger or smaller than in the Standard Model. The branch with larger values of $R_{{\bar{b}}b}$ is also related to the variation of the Higgs width, while the points corresponding to a decrease of both ratios are due to an enhancement of decays to light SUSY particles [43–45].^7^

**Fig. 13:**
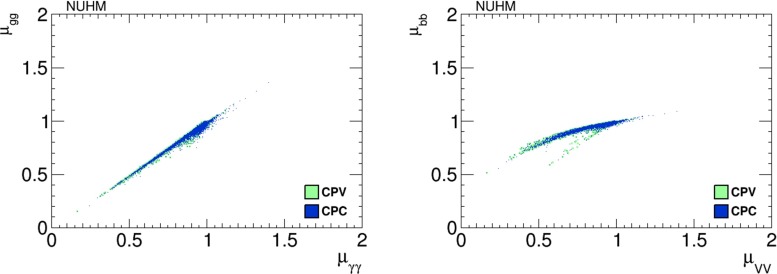
Scatter plots of the $h_1$ signal strengths in the NUHM2 scenario in the CP-violating limit $\Phi _\alpha = 0$ (*blue dots*) and in the CP-violating sample (*green dots*). The *left panel* displays a strong linear correlation between $\mu _{\gamma \gamma }$ and $\mu _{gg}$, and the *right panel* displays a bimodal correlation between $\mu _{VV}$ and $\mu _{{\bar{b}}b}$

Scatter plots of $h_1$ signal strengths $\mu _X$ in the NUHM2 scenario with the CP-violating phases $\Phi _\alpha =0$ (blue dots) and $\ne 0$ (green dots) are shown in Fig. 13.

We see a strong, almost linear correlation between $\mu _{\gamma \gamma }$ and $\mu _{gg}$ in the left panel, and in the right panel we see a correlation between $\mu _{VV}$ and $\mu _{{\bar{b}}b}$ that is bimodal for small values of $\mu _{VV}$. No significant difference is observed between the CP-conserving and the CP-violating cases.

**Fig. 14:**
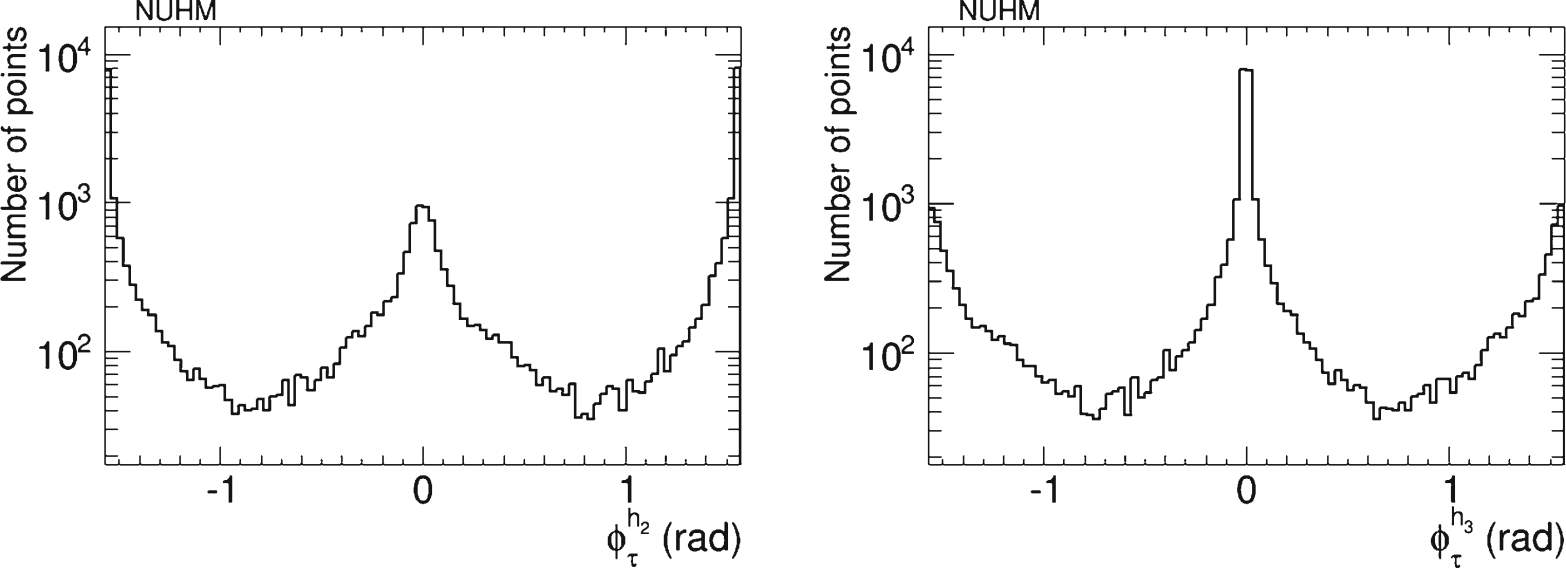
The distributions of (*left*) the CP-violating phase $\phi _\tau ^{h_2}$ in $h_2 \tau \tau$ couplings and (*right*) the CP-violating phase $\phi _\tau ^{h_3}$ in $h_3 \tau \tau$ couplings in the NUHM2 scenario (8), found after applying all the constraints using the geometric approach described in the text

**Fig. 15:**
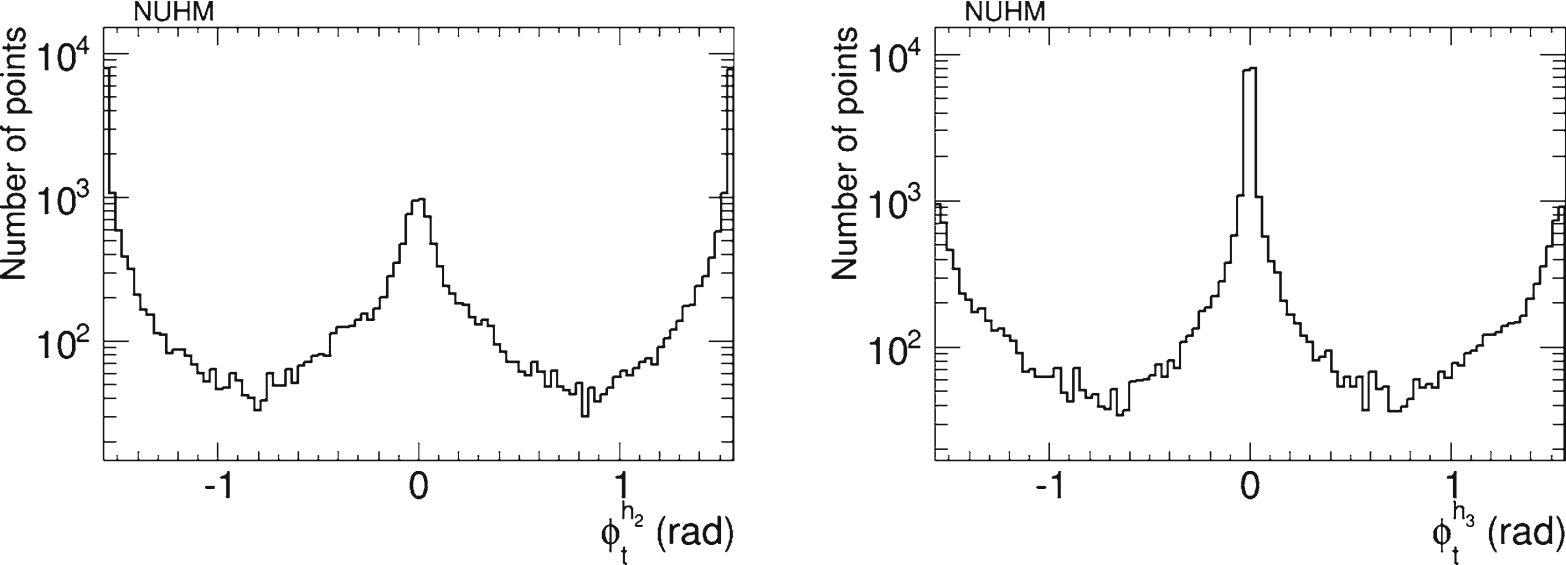
The distributions of (*left*) the CP-violating phase $\phi _t^{h_2}$ in $h_2 {\bar{t}} t$ couplings and (*right*) the CP-violating phase $\phi _t^{h_3}$ in $h_3 {\bar{t}} t$ couplings in the NUHM2 scenario (8), found after applying all the constraints using the geometric approach described in the text

The prospects for CP violation in the couplings of the heavy neutral Higgs bosons to $\tau ^+ \tau ^-$ and ${\bar{t}} t$ in the NUHM2 scenario (8) are shown in Figs. 14 and 15. As in the CMSSM case discussed previously, we find that after imposing all the constraints the phases for the $h_1$ couplings are small, namely ${\lesssim } 0.02$ radians. On the other hand, $h_{2,3}$ decays may provide interesting prospects for probing CP violation also in this NUHM2 scenario.

### CPX

We now apply the iterative geometric approach with four EDMs and one CP-violating observable described earlier to a CPX scenario in which

9 $$\begin{aligned}&M_{Q_3} =M_{U_3} =M_{D_3}=M_{L_3}=M_{E_3}\equiv M_S, \nonumber \\&\mu =4M_S,\quad |A_{t,b,\tau }|=2 M_S,\quad |M_{1,2}|=1~\mathrm{TeV},\nonumber \\&\quad |M_3|=3~\mathrm{TeV}, \end{aligned}$$

performing random scans over the following parameter ranges:

10 $$\begin{aligned}&M_S \in [50,3000]~\mathrm{GeV},\quad m_{H^\pm }\in [1,2000]~\mathrm{GeV},\nonumber \\&\quad \tan \beta \in [1,60], \end{aligned}$$

with the six CP-violating phases of the MCPMFV model being considered independent, as before.

**Fig. 16:**
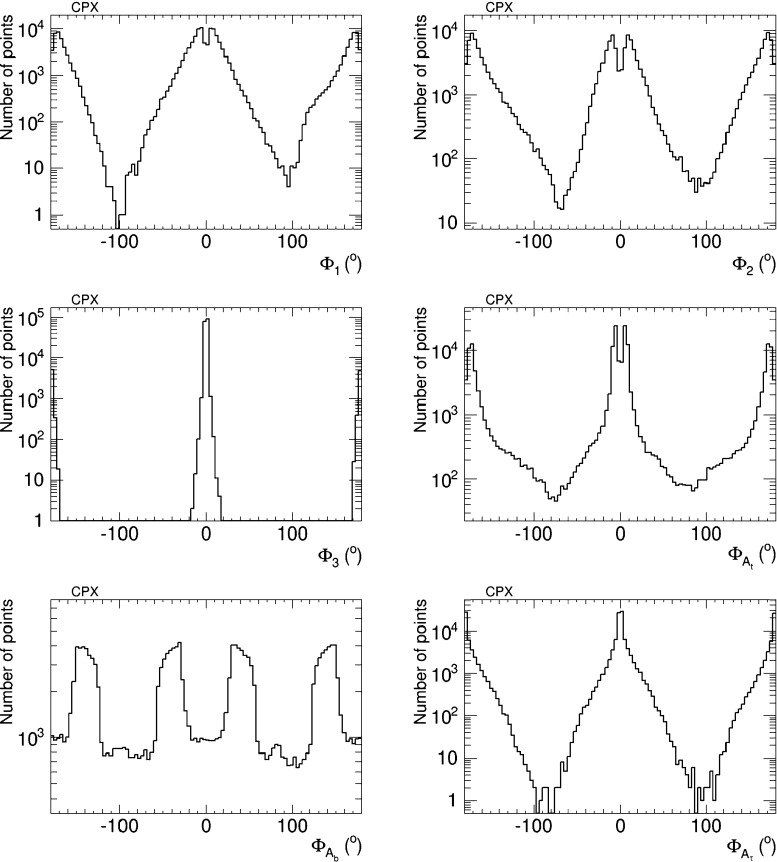
Sampling of the CP-violating phases $\Phi _\alpha$ in the CPX scenario generated in the iterative geometric approach, imposing the EDM and other constraints

Figure 16 displays the distributions of the six CP-violating phases $\Phi _\alpha$ sampled in our analysis. We emphasise that these distributions do not have any ‘probability’ or ‘likelihood’ interpretation. Rather, they serve to indicate how well our iterative geometric procedure gives access to large values of the phases that are difficult to sample in a simple random scan, because of the cancellations required to bring the EDMs within the allowed ranges shown in Table 1. We see that the effectiveness of the procedure differs significantly for different phases. For example, in the case of $\Phi _{A_b}$ our procedure yields almost as many parameter sets with $\Phi _{A_b} \sim {\pm } 90^{\circ }$ as with $\Phi _{A_b} \sim 0^{\circ }$ or $180^{\circ }$, and actually yields *more* parameter sets with intermediate values of $\Phi _{A_b}$. In the case of $\Phi _{A_t}$, the procedure yields a factor ${\sim } 100$ lower sampling density for $\Phi _{A_b} \sim {\pm } 90^{\circ }$ than for $\Phi _{A_b} \sim 0^{\circ }, 180^{\circ }$, and larger factors for $\Phi _2$, $\Phi _1$ and $\Phi _{A_\tau }$. Finally, we find no parameter sets for $\Phi _3 \sim \pm 90^{\circ }$: this is because (for the choices of soft supersymmetry-breaking parameters in (9)) there is no way to cancel the contributions of this and the other phases to all the EDMs simultaneously.

**Fig. 17:**
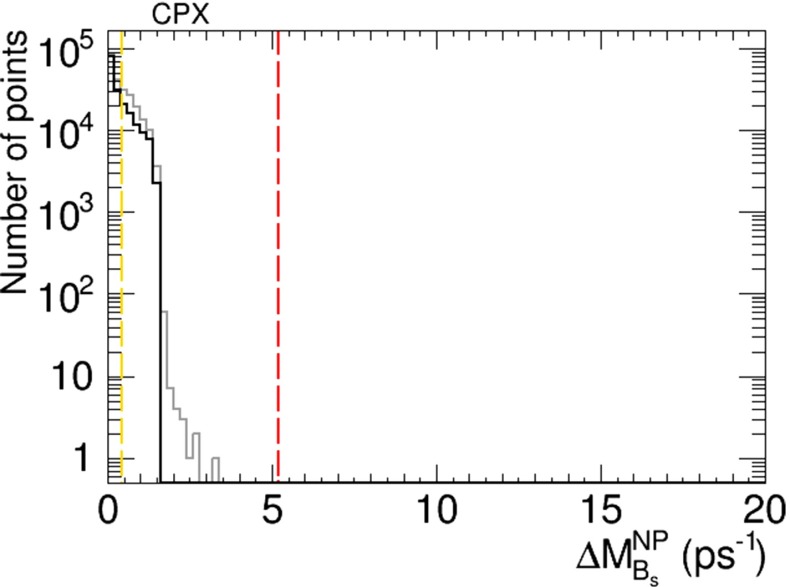
Histogram of the possible new physics contribution to $B_s$ mixing, $\Delta M^\mathrm{NP}_{B_s}$, in the CPX scenario. The *grey histogram* is for points satisfying the Higgs mass and other constraints, and the *black histogram* is for points that also satisfy the EDM constraints. The *vertical red dashed line* is the present experimental upper limit on $\Delta M^\mathrm{NP}_{B_s}$, and the *vertical yellow dashed line* shows the potential of a reduction in the current theoretical uncertainty in the Standard Model by a factor of 10

In the CPX scenario we do not find values of $A_\mathrm{CP}$ that are large enough to be observable in the foreseeable future. However, we do find a possible signature in the new physics contribution to $B_s$ meson mass mixing, $\Delta M^\mathrm{NP}_{B_s}$, as shown in Fig. 17. The grey histogram is for CPX points satisfying the Higgs mass and other constraints, and the black histogram is for points that also satisfy the EDM constraints, including the present experimental upper limit on $\Delta M^\mathrm{NP}_{B_s}$, which is shown as the vertical red dashed line. The magnitude of this upper limit is largely due to the theoretical uncertainty in the Standard Model contribution to $B_s$ mixing, which is in turn associated with lattice calculations. If this uncertainty could be reduced by a factor of 10, the sensitivity to new physics in $B_s$ mixing would become that indicated by the vertical yellow dashed line in Fig. 17, which could explore many of the CPX models indicated by the black histogram.

**Fig. 18:**
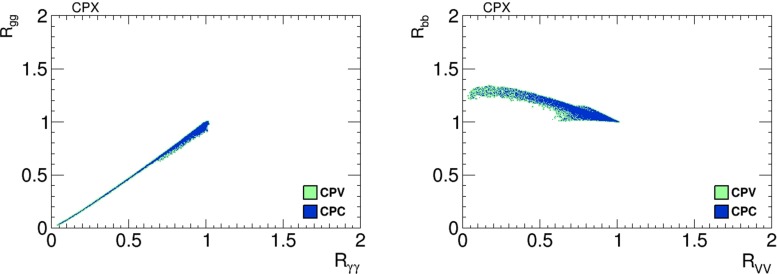
Scatter plots of branching ratios, normalised to the Standard Model values, for decays of the lightest Higgs boson, $h_1$, in the CPX scenario in the CP-violating limit $\Phi _\alpha = 0$ (*blue dots*) and in the CP-violating sample (*green dots*). The *left panel* displays a linear correlation between $R_{\gamma \gamma }$ and $R_{gg}$, and the *right panel* displays a nonlinear anti-correlation between $R_{VV}$ and $R_{{\bar{b}}b}$

We display in Fig. 18 scatter plots of values of branching ratios in the CPX scenario of the lightest Higgs boson, $h_1$, normalised relative to the Standard Model values. The left panel shows $(R_{\gamma \gamma }, R_{gg})$ and the right panel shows $(R_{VV}, R_{{\bar{b}}b})$ in the limits where the phases $\Phi _\alpha = 0$ (blue dots) and scanning over the values of $\Phi _\alpha \ne 0$ allowed by the EDMs (green dots). There are very small differences between the values of these quantities found in the CP-conserving and CP-violating samples. In both cases, correlated substantial reductions in $R_{\gamma \gamma }$ and $R_{gg}$ are possible, as is a large reduction in $R_{VV}$ relative to the Standard Model value. On the other hand, the ‘Cuba’-shaped plot in the right panel shows that $R_{{\bar{b}}b}$ is anti-correlated with $R_{VV}$, and may be enhanced to ${\sim } 1.3$ times the Standard Model value.

**Fig. 19:**
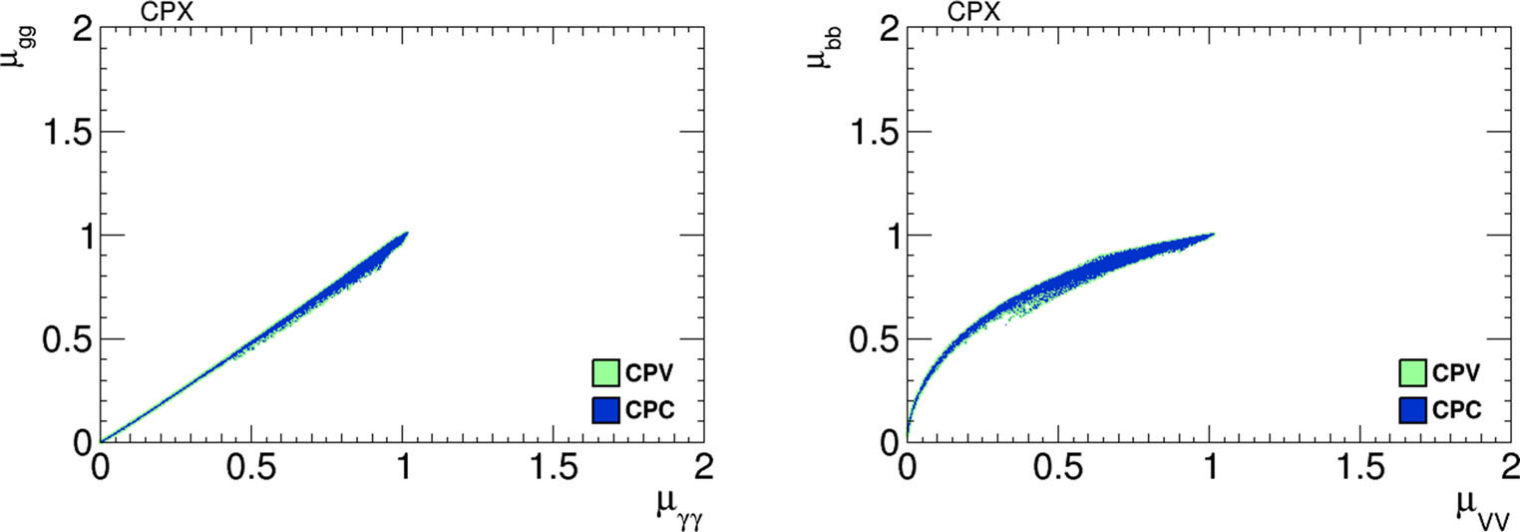
Scatter plots of the $h_1$ signal strengths in the CPX scenario in the CP-violating limit $\Phi _\alpha = 0$ (*blue dots*) and in the CP-violating sample (*green dots*). The *left panel* displays a strong linear correlation between $\mu _{\gamma \gamma }$ and $\mu _{gg}$, and the *right panel* displays a nonlinear correlation between $\mu _{VV}$ and $\mu _{{\bar{b}}b}$

Figure 19 shows scatter plots of $h_1$ signal strengths $\mu _X$ in the CPX scenario with the CP-violating phases $\Phi _\alpha =0$ (blue dots) and ${\ne } 0$ (green dots): again only very small differences are seen. In the left panel we see a strong, almost linear correlation between $\mu _{\gamma \gamma }$ and $\mu _{gg}$, and in the right panel we see a nonlinear correlation between $\mu _{VV}$ and $\mu _{{\bar{b}}b}$.

As already mentioned, our results for $A_\mathrm{CP}$ in the CPX scenario are very small, so we do not display them. Taken together with the results shown in Figs. 18 and 19, where no distinctive signatures of non-zero phases $\Phi _\alpha \ne 0$ are visible, our results suggest that one should look elsewhere for probes of CP violation in the CPX scenario.

**Fig. 20:**
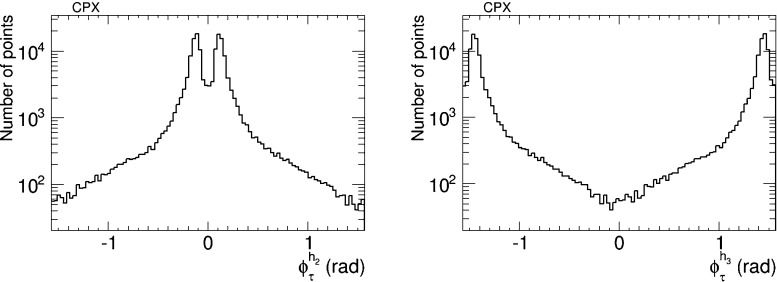
The distributions of (*left*) the CP-violating phase $\phi _\tau ^{h_2}$ in $h_2 \tau \tau$ couplings and (*right*) the CP-violating phase $\phi _\tau ^{h_2}$ in $h_2 \tau \tau$ couplings in the CPX scenario (9, 10), found after applying all the constraints using the geometric approach described in the text

**Fig. 21:**
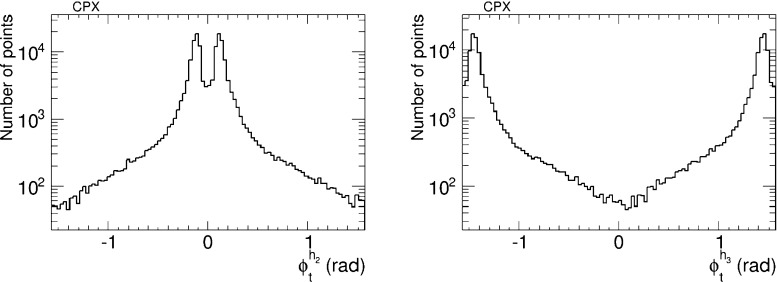
The distributions of (*left*) the CP-violating phase $\phi _t^{h_2}$ in $h_2 {\bar{t}} t$ couplings and (*right*) the CP-violating phase $\phi _t^{h_2}$ in $h_2 {\bar{t}} t$ couplings in the CPX scenario (9, 10), found after applying all the constraints using the geometric approach described in the text

We have also analysed the prospects for CP violation in the couplings of the neutral Higgs bosons to $\tau ^+ \tau ^-$ and ${\bar{t}} t$ in the CPX scenario (9, 10), as given by the phases $\phi ^{h_i}_\tau$ and $\phi ^{h_i}_t$ for $i = 1, 2, 3$ defined in (7). As in the CMSSM case discussed previously, we find that after imposing the EDM constraints the phases for the $h_1$ couplings are small, $\phi ^{h_i}_\tau \lesssim 0.1$ radians and $\phi ^{h_i}_t \lesssim 0.02$ radians. On the other hand, the phases for the $h_2$ and $h_3$ couplings may again be quite large, as seen in Figs. 20 and 21, respectively. Thus $h_{2,3}$ decays may also provide interesting prospects for probing CP violation in this CPX scenario.

### Phenomenological MSSM (pMSSM)

We now consider the MCPMFV version of the phenomenological MSSM (pMSSM), which has 25 parameters: the 19 real parameters

11 $$\begin{aligned}&M_{1,2,3},\; M_{{\tilde{Q}_L}, {\tilde{U}_R}, {\tilde{D}_R}, {\tilde{L}_L}, {\tilde{E}_R}},\; M_{{\tilde{Q}_{3L}}, {\tilde{t}_R}, {\tilde{b}_R}, {\tilde{L}_{3L}}, {\tilde{\tau }_R}}, \nonumber \\&\quad M_{H^\pm },\mu , \tan \beta ,\; A_{t, b, \tau } \end{aligned}$$

and the six phases $\Phi _\alpha$ discussed previously. We perform a scan of the pMSSM parameter space using the iterative geometric approach described in Sect. 2. We first generated about 40 million points, and then kept only points with a neutral Higgs boson with a mass in the range 121–129 GeV (thereby allowing for a conservative theoretical uncertainty in the Higgs mass calculation), and with a neutralino LSP. These requirements reduced the number of points to about 1 million. Imposing the EDM constraints then left about 150000 valid points. In the following plots, in addition to these constraints, we also impose flavour constraints, the cosmological upper bound on the dark matter density, the LUX direct upper limit on spin-independent dark matter scattering (except when the same observable is plotted), and we require squarks and the gluino to have masses above 500 GeV.

**Fig. 22:**
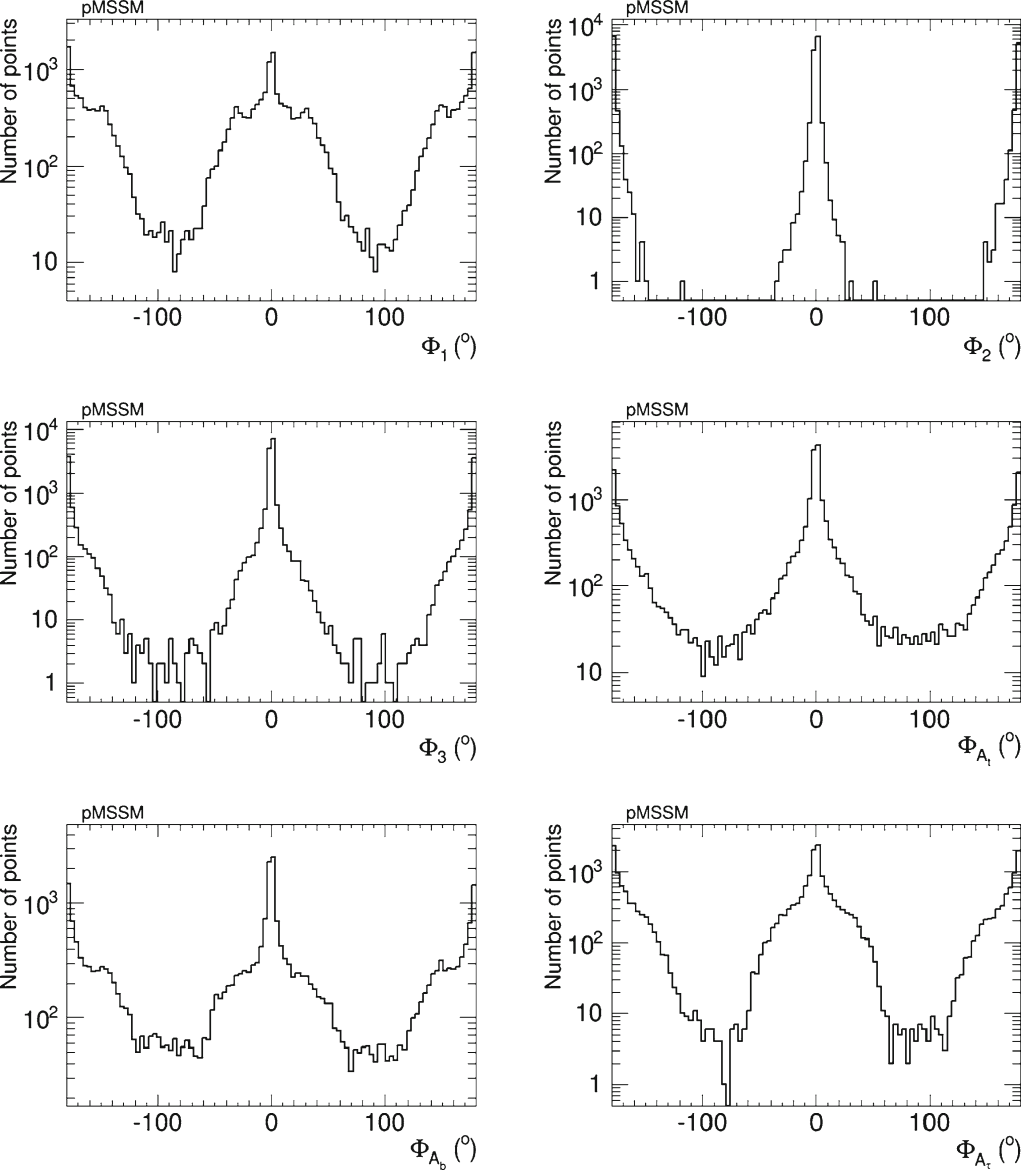
Sampling of the CP-violating phases $\Phi _\alpha$ in the pMSSM scenario generated in the iterative geometric approach, imposing the EDM and other constraints

Figure 22 shows the samplings of the phases $\Phi _\alpha$ obtained after imposing these constraints. We see that values of $\Phi _{A_{t,b}}$ and $\Phi _1 \sim {\pm } 90^{\circ }$ are quite well sampled, as are values of $\Phi _{A_\tau } \sim 90^{\circ }$. On the other hand, large values of $\Phi _3$ are less well sampled, and the range of $\Phi _2$ is very restricted with only small deviations from the CP-conserving cases being allowed.

**Fig. 23:**
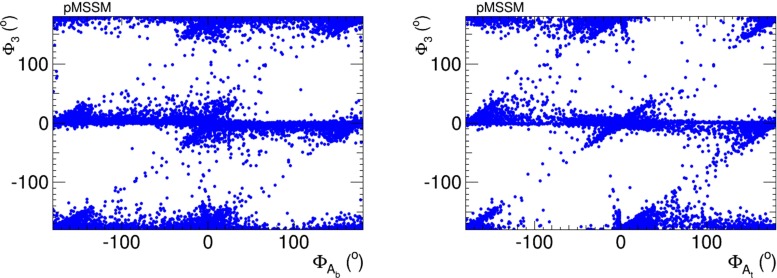
Correlations of $\Phi _3$ with $\Phi _\mathrm{{A_b}}$ (*left panel*) and $\Phi _3$ with $\Phi _\mathrm{{A_t}}$ (*right panel*) imposed by the EDM constraints in the pMSSM scenario

We see in Fig. 23 the extent to which the EDM constraints impose cancellations $\Phi _3$ and $\Phi _{A_t}$ (left panel) and between $\Phi _3$ and $\Phi _{A_b}$ (right panel). We see that large values of $(\Phi _{A_t, A_b}, \Phi _3) \sim ( {\pm }90^{\circ }, {\pm }90^{\circ })$ are allowed, and we also see diagonal features corresponding to correlations. As in the NUHM2, it is apparent that the EDM constraints do not require all the CP-violating phases to be small simultaneously.

**Fig. 24:**
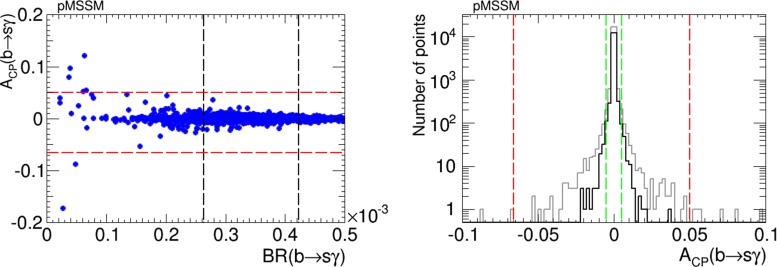
*Left panel* Scatter plot of the branching ratio for $b\rightarrow s\gamma$ decay versus its CP-violating asymmetry, $A_\mathrm{CP}$, in the pMSSM scenario. The *vertical black dashed lines* represent the allowed range for the $b \rightarrow s \gamma$ branching ratio, and the *horizontal red dashed lines* represent the present experimental limits on $A_\mathrm{CP}$. *Right panel* Histogram of $A_\mathrm{CP}$ in the pMSSM, imposing only the Higgs mass and EDM cuts (*grey* full sample, *black* points satisfying the EDM constraints). The *vertical red dashed lines* represent the present experimental limits, and the *vertical green dashed lines* represent the prospective future improvement in the sensitivity to $A_\mathrm{CP}$ by a factor of 10

The left panel of Fig. 24 displays a scatter plot of the values of $A_\mathrm{CP}$ found in the pMSSM using the iterative geometric approach. We see that values ${\lesssim }3$ % are possible for values of the $b \rightarrow s \gamma$ branching ratio lying within the experimentally allowed range. The right panel of Fig. 24 shows a histogram of $A_\mathrm{CP}$ values, imposing only the Higgs mass and EDM cuts. Here we see tails extending to larger values of $|A_\mathrm{CP}|$ that lie outside the experimentally allowed range when the EDM constraints are not applied (grey histogram), whereas the black histogram is for points satisfying the EDM constraints. The vertical red dashed lines show the present experimental constraints on $A_\mathrm{CP}$, and the possible future improvement in the experimental sensitivity by a factor of 10 is indicated by vertical green dashed lines. As in the NUHM2, there are CP-violating pMSSM parameter sets that could be explored with such an improvement: it would provide additional information on CP violation within the pMSSM.

**Fig. 25:**
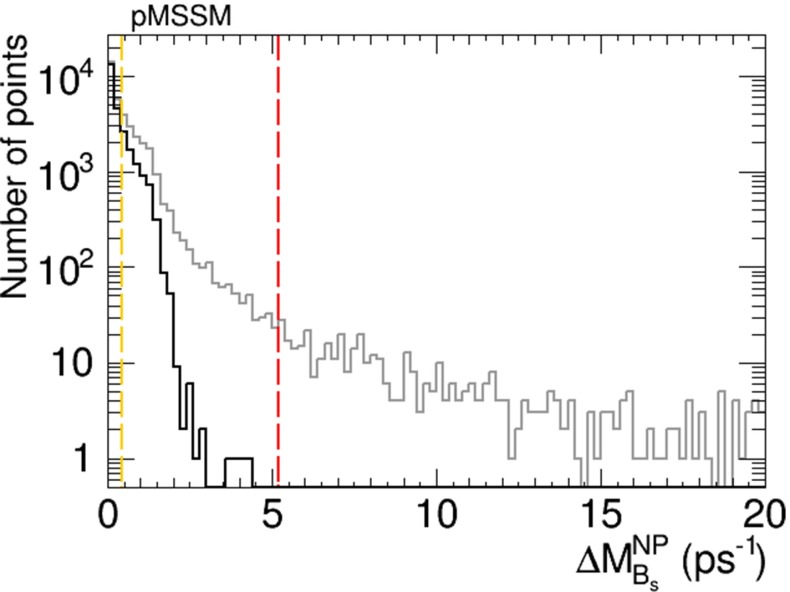
Histogram of the possible new physics contribution to $B_s$ mixing, $\Delta M^\mathrm{NP}_{B_s}$, in the pMSSM scenario. The *grey histogram* is for points satisfying the Higgs mass and other constraints, and the *black histogram* is for points that also satisfy the EDM constraints. The *vertical red dashed line* is the present experimental upper limit on $\Delta M^\mathrm{NP}_{B_s}$, and the *vertical yellow dashed line* shows the potential of a reduction in the current theoretical uncertainty in the Standard Model by a factor of 10

The possible new physics contribution to $B_s$ meson mass mixing, $\Delta M^\mathrm{NP}_{B_s}$, in the pMSSM scenario is shown in Fig. 25. As in the previous cases studied, the grey histogram is for the full sample, and the black histogram is for points that also satisfy the EDM constraints. If the theoretical uncertainty in the Standard Model contribution to $B_s$ mixing could be reduced by a factor of 10 thanks to improved lattice calculations, the sensitivity to $\Delta M^\mathrm{NP}_{B_s}$ would become that indicated by the vertical yellow dashed line in Fig. 25. In this case, many of the pMSSM models that are currently viable (indicated by the black histogram) could be explored.

**Fig. 26:**
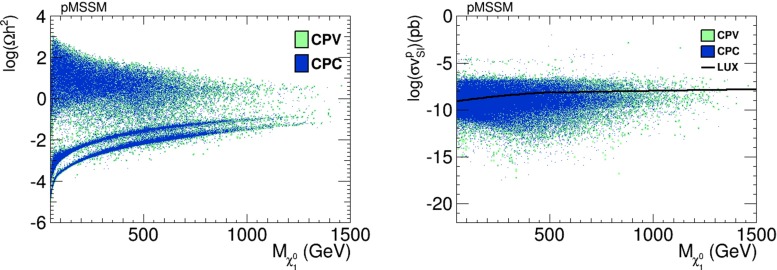
*Left panel* Scatter plot of the dark matter relic density as a function of the neutralino mass in the pMSSM scenario. *Right panel* Scatter plot of the spin-independent dark matter scattering cross section $\sigma _\mathrm{SI}^p v$ as a function of the neutralino mass in the pMSSM scenario. In both panels, CP-conserving parameter choices are denoted by *blue dots*, and CP-violating parameter choices by *green dots*

In Fig. 26, we show in the left panel the values of the relic LSP density $\Omega _{\chi } h^2$ that we find in our pMSSM scan, and in right panel we show values of the spin-independent dark matter scattering cross section $\sigma _\mathrm{SI}^p v$. We see that values of $\Omega _{\chi } h^2$ considerably above the cosmological upper limit are possible in both the CP-conserving (blue dots) and the CP-violating cases (green dots). We also see in the right panel of Fig. 26 that values of $\sigma _\mathrm{SI}^p v$ above the LUX upper limit are also possible. In both panels, there are no large differences between the CP-conserving and CP-violating cases.

**Fig. 27:**
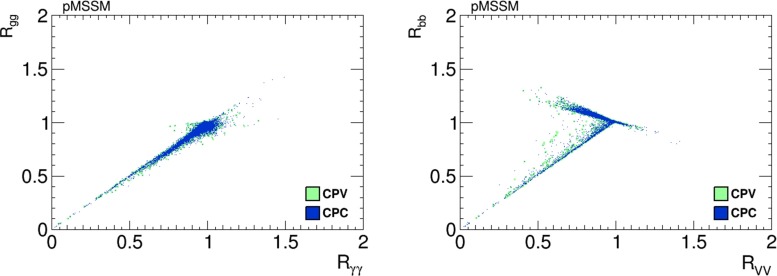
Scatter plots of branching ratios, normalised to the Standard Model values, for decays of the lightest Higgs boson, $h_1$, in the pMSSM scenario in the CP-violating limit $\Phi _\alpha = 0$ (*blue dots*) and in the CP-violating sample (*green dots*). The *left panel* displays a strong correlation between $R_{\gamma \gamma }$ and $R_{gg}$, and the *right panel* displays a bimodal correlation between $R_{VV}$ and $R_{{\bar{b}}b}$

Scatter plots of values of $h_1$ branching ratios in the pMSSM scenario are in Fig. 27, the left panel displaying $(R_{\gamma \gamma }, R_{gg})$ and the right panel displaying $(R_{VV}, R_{{\bar{b}}b})$. As previously, the blue dots are CP-conserving parameter choices with $\Phi _\alpha = 0$, and the green dots are from a scan of CP-violating points with $\Phi _\alpha \ne 0$.

As in the NUHM2 scenario, we note in the left panel a strong correlation between $R_{\gamma \gamma }$ and $R_{gg}$, which may be either much smaller than in the Standard Model or somewhat larger, and we also see in the right panel that a large reduction in $R_{VV}$ is possible. Also as in the NUHM2 scenario, the reduction in $R_{VV}$ may be accompanied by values of $R_{{\bar{b}}b}$ that are either larger or smaller than in the Standard Model, the latter possibility arising when the Higgs boson can decay into light sparticles.

**Fig. 28:**
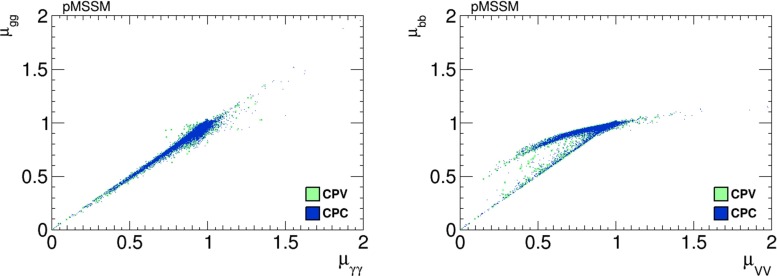
Scatter plots of the $h_1$ signal strengths in the pMSSM scenario in the CP-violating limit $\Phi _\alpha = 0$ (*blue dots*) and in the CP-violating sample (*green dots*). The *left panel* displays a strong linear correlation between $\mu _{\gamma \gamma }$ and $\mu _{gg}$, and the *right panel* displays a bimodal correlation between $\mu _{VV}$ and $\mu _{{\bar{b}}b}$ for smaller values

Figure 28 displays scatter plots of $h_1$ signal strengths $\mu _X$ in the pMSSM scenario in the CP-conserving case with phases $\Phi _\alpha =0$ (blue dots) and in the CP-violating case where the $\Phi _\alpha \ne 0$ (green dots). As in the NUHM2 case, we see a strong correlation between $\mu _{\gamma \gamma }$ and $\mu _{gg}$ in the left panel, and in the right panel we see a correlation between $\mu _{VV}$ and $\mu _{{\bar{b}}b}$ that becomes bimodal for small values of $\mu _{VV}$.

**Fig. 29:**
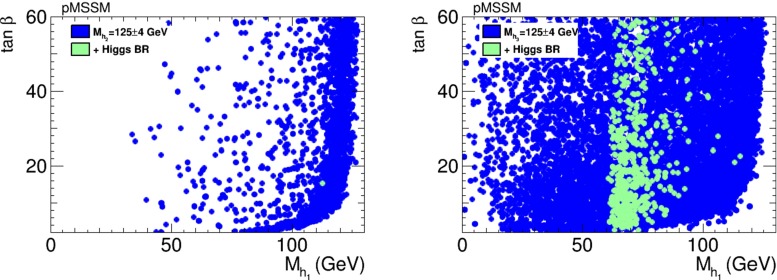
Scatter plots of pMSSM points in the ($m_{h_1},\tan \beta$) plane in the case where either the $h_2$ (*left panel*) or the $h_3$ (*right panel*) is the Higgs boson discovered at the LHC, applying only the EDM and Higgs mass constraints (*blue dots*), and applying also the Higgs signal strength constraints (*green dots*). We find no points that satisfy in addition the neutral and charged heavy Higgs search constraints

**Fig. 30:**
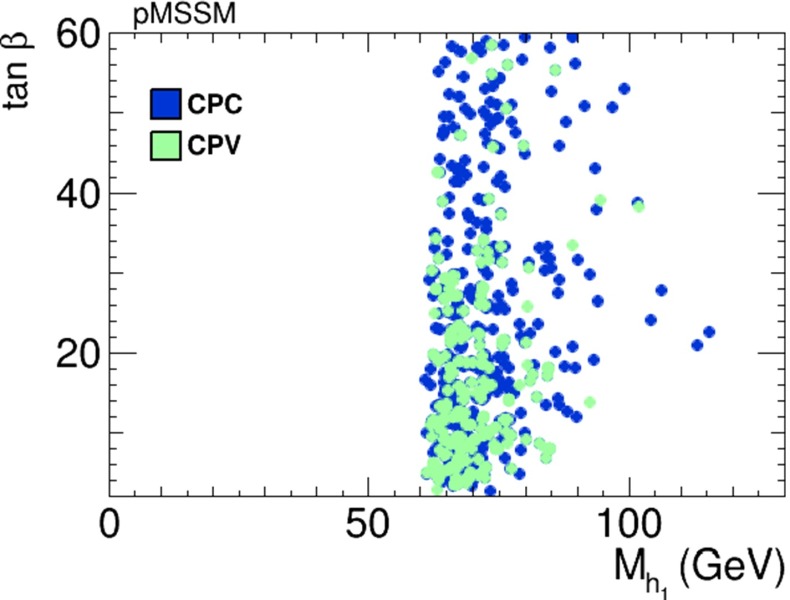
Scatter plot of pMSSM points in the ($m_{h_1},\tan \beta$) plane in the case where the Higgs boson discovered at the LHC is identified as the $h_3$, for the points satisfying the Higgs signal strength constraints as well as the EDM constraints

We have also studied whether the Higgs boson discovered at the LHC might be one of the heavier Higgs bosons in the pMSSM, with or without CP violation. As seen in the left panel of Fig. 29, if the known Higgs boson is identified with the $h_2$, it is not possible to satisfy the Higgs signal strength constraints. This is possible if the discovered Higgs boson is identified with the $h_3$, as seen (green dots) in the right panel of Fig. 29, in which case the $h_1$ mass is about 60–80 GeV. Figure 30 displays these points in both the CP-conserving case (blue dots) and the CP-violating case (green dots), which are quite similar. On the other hand, none of these points survive the charged Higgs and $A/H \rightarrow \tau \tau$ constraints, nor the flavour constraints. We therefore conclude that the pMSSM does not provide a way to conceal a neutral Higgs boson that is lighter than the one discovered, even if CP is violated.

**Fig. 31:**
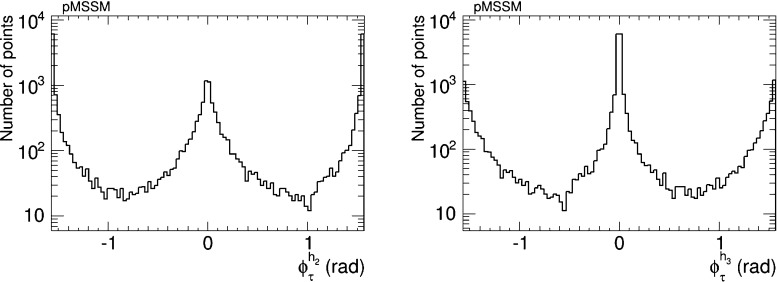
The distributions of (*left*) the CP-violating phase $\phi _\tau ^{h_2}$ in $h_2 \tau \tau$ couplings and (*right*) the CP-violating phase $\phi _\tau ^{h_3}$ in $h_3 \tau \tau$ couplings in the pMSSM scenario (11), found after applying all the constraints using the geometric approach described in the text

**Fig. 32:**
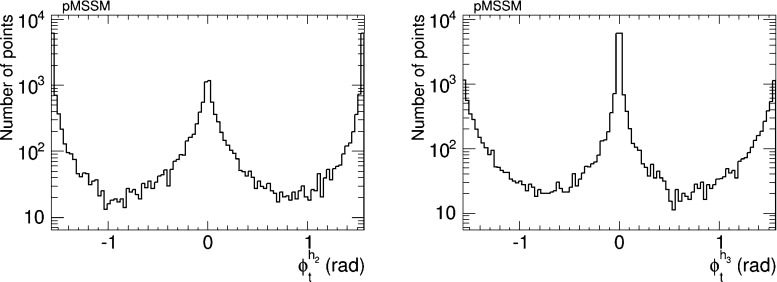
The distributions of (*left*) the CP-violating phase $\phi _t^{h_2}$ in $h_2 {\bar{t}} t$ couplings and (*right*) the CP-violating phase $\phi _t^{h_3}$ in $h_3 {\bar{t}} t$ couplings in the pMSSM scenario (11), found after applying all the constraints using the geometric approach described in the text

Assuming that the Higgs boson discovered at the LHC is indeed the lightest MSSM Higgs boson $h_1$, we now assess the prospects for CP violation in the couplings of the heavy neutral Higgs bosons to $\tau ^+ \tau ^-$ and ${\bar{t}} t$ in the pMSSM scenario (11) which are shown in Figs. 31 and 32. We see that, as in the CMSSM, CPX and NUHM2 cases discussed previously, $h_{2,3}$ decays may provide interesting prospects for probing CP violation also in this pMSSM scenario. On the other hand, we again find that after imposing all the constraints the phases for the $h_1$ couplings are small, namely $\phi ^{h_1}_\tau \lesssim 0.03$ radians and $\phi ^{h_1}_t \lesssim 0.02$ radians, respectively.

## Conclusions

The geometrical approach to implementing EDM constraints and maximizing other CP-violating observables proposed in [2] provides a suitable way to explore the possibilities for CP violation in variants of the MSSM, which we have applied in this paper to explore the CMSSM, the CPX scenario, the NUHM2 and the pMSSM. We have adopted an iterative extension of the geometric approach, which is suitable for exploring larger values of the CP-violating phases. Our explorations have been within the maximally CP-violating, minimal flavour-violating (MCPMFV) framework with six CP-violating phases, of which two combinations are unconstrained a priori by the four EDM constraints. The following are our principal results:

- In the CMSSM we have explored CP-violating generalisations of the low-mass best-fit point (6) that was identified in [11, 12], where we found relatively little scope for large deviations from the CP-conserving case, e.g., in the masses of the Higgs bosons and the spin-independent dark matter scattering cross section. Moreover, we found that only very small values of $A_\mathrm{CP} \lesssim 0.001$ would be possible in this case, and the new physics contribution to $B_s$ meson mixing, $\Delta M^\mathrm{NP}_{B_s}$, would not be observable.
- We have then explored the CPX scenario (9), where we also found no scope for measurable values of $A_\mathrm{CP}$. On the other hand, we found in this model that $\Delta M^\mathrm{NP}_{B_s}$ could be large enough to provide a possible signature if the current lattice theoretical uncertainty in the Standard Model contribution to $B_s$ mixing could be reduced by a factor of 10, as seen in Fig. 17.
- The situation in the NUHM2 scenario (8) is rather more favourable for observable signals of CP violation. In this case, $A_\mathrm{CP}$ could be as large as ${\sim } 2$ % and hence lie well within the reach of experiment, as seen in Fig. 9, and $\Delta M^\mathrm{NP}_{B_s}$ might also be large enough to provide a possible experimental signature, as seen in Fig. 10.
- A similar situation was found in the pMSSM scenario (11), in which case $A_\mathrm{CP}$ could be as large as ${\sim } 3$ %, as seen in Fig. 24, again within the reach of experiment. We also find in this scenario that $\Delta M^\mathrm{NP}_{B_s}$ could be large enough to be observable with a prospective reduction in the theoretical uncertainty in the Standard Model calculation of $B_s$ mixing, as seen in Fig. 25.
- In all the scenarios studied, the CP-violating phases in the $h_1 \tau ^+ \tau ^-$ and $h_1 {\bar{t}} t$ couplings are small. On the other hand, the phases in the $h_{2,3} \tau ^+ \tau ^-$ and $h_{2,3} {\bar{t}} t$ couplings can be quite large, and may present interesting prospects for future $pp$, $e^+ e^-$ and $\mu ^+ \mu ^-$ experiments, though their detailed study lies beyond the scope of this work.

Our analysis serves as a reminder that the EDM constraints do not force all the six non-KM CP-violating phases in MCPMFV to be small, and that in some variants of the MSSM there could be observable signatures of CP violation beyond the Standard Model, e.g., $A_\mathrm{CP}$ in $b \rightarrow s \gamma$ decay. We look forward to a generation of $A_\mathrm{CP}$ measurements, and also to improved theoretical calculations of the Standard Model contribution to $B_s$ meson mixing, which might enable a new physics contribution $\Delta M^\mathrm{NP}_{B_s}$ to be isolated. If enough soft supersymmetry-breaking parameters could be measured, and both $A_\mathrm{CP}$ and $\Delta M^\mathrm{NP}_{B_s}$ could be shown to have measurable deviations from the Standard Model, one might finally be able to fix all the six non-KM CP-violating phases in MCPMFV.

## Footnotes

1 We assume that the strong CP phase is negligible, and also neglect the phases in the trilinear couplings of the sfermions in the first and second generations, which are much less important for phenomenology.

2 We note in passing that there are also well-motivated supersymmetric models in which the phases are naturally small, so that the EDM bounds are not very constraining, see [5] for example.

3 Partial results in the case of CMSSM were presented in [15].

4 We use Latin indices $i,j,\ldots$ for the EDMs, and Greek indices $\alpha ,\beta ,\ldots$ for the CP-violating phases.

5 This analysis also found a high-mass best-fit point with a slightly lower value of the global $\chi ^2$ function. However, the ATLAS jets + missing transverse energy constraint has subsequently been revised, and the low-mass point is now the global minimum of the $\chi ^2$ function [12].

6 Small values of $R_{\gamma \gamma }$ and $R_{gg}$ are disfavoured by LHC Higgs measurements.

7 Small values of $R_{VV}$ are disfavoured by LHC Higgs measurements, whereas a relatively large range of $R_{{\bar{b}}b}$ is still allowed.
